# Proteomic Profiling of Tears in Blau Syndrome Patients in Identification of Potential Disease Biomarkers

**DOI:** 10.3390/ijms25158387

**Published:** 2024-08-01

**Authors:** Paola Galozzi, Sara Bindoli, Chiara Baggio, Ilaria Battisti, Andrea Leonardi, Daniela Basso, Giorgio Arrigoni, Paolo Sfriso

**Affiliations:** 1 Laboratory Medicine Unit, Department of Medicine DIMED, University of Padova, Via Giustiniani 2, 35128 Padova, Italy; 2 Rheumatology Unit, Department of Medicine DIMED, University of Padova, Via Giustiniani 2, 35128 Padova, Italy; 3 Department of Biomedical Sciences, University of Padova, 35128 Padova, Italy; 4 Ophthalmology Unit, Department of Neuroscience, University of Padova, 35128 Padova, Italy

**Keywords:** tear fluids, biomarkers, proteomics, Blau syndrome, autoinflammatory disease, uveitis

## Abstract

Blau syndrome (BS) is a rare autoinflammatory granulomatosis characterized by granulomatous arthritis, uveitis, and dermatitis. Ocular complications are particularly severe in BS, significantly contributing to morbidity. This study aims to identify potential biomarkers for BS ocular degeneration through proteomic profiling of tear samples from affected patients. Seven subjects from the same family, including four carriers of the BS-associated *NOD2* mutation (p.E383K), were recruited alongside healthy controls. Tear samples were collected using Schirmer strips and analyzed via mass spectrometry. A total of 387 proteins were identified, with significant differences in protein expression between BS patients, healthy familial subjects, and healthy controls. Key findings include the overexpression of alpha-2-macroglobulin (A2M) and immunoglobulin heavy constant gamma 4 (IGHG4) in BS patients. Bioinformatic analysis revealed that differentially expressed proteins are involved in acute-phase response, extracellular exosome formation, and protein binding. Notably, neutrophils’ azurophilic granule components, as azurocidin (AZU1), myeloperoxidases (MPO), and defensins (DEFA3), were highly expressed in the most severely affected subject, suggesting a potential role of neutrophils in BS ocular severity. These proteins might be promising biomarkers for ocular involvement in BS, facilitating early detection and tailored treatment strategies.

## 1. Introduction

Blau syndrome (BS) stands out as a remarkably uncommon autosomal dominant inherited autoinflammatory disorder, manifesting in approximately 0.06 to 1 per 100,000 individuals within the general population [1]. BS represents a rare autoinflammatory granulomatosis, characterized by a spectrum of clinical manifestations primarily affecting the skin, joints, and eyes. The etiology of this disease involves mutations in the nucleotide-binding oligomerization domain-containing 2 (*NOD2*) gene situated on chromosome 16 [2]. *NOD2* mutations found in BS lead to a spontaneous oligomerization of the protein. As a result, mutated NOD2 leads to NF-κB hyper-activation, particularly when stimulated by bacterial wall components [3]. The most frequently observed mutations are p.R334W and p.R334Q, but several other variants are reported within the central ATPase domains, such as p.E383K, where the glutamic acid at codon 383, which is acidic and polar, was replaced with lysine, which is basic and polar. This variant has been reported as associated with BS and observed to co-segregate with the disease in affected family members [4,5,6,7,8]. Experimental studies have supported the pathogenicity of the mutation, showing that this missense change affects NOD2 function and is expected to disrupt NOD2 protein function [2,9].

Clinically, BS is characterized by a symptomatic triad comprising granulomatous arthritis, uveitis, and dermatitis [10]. The arthritis frequently involves multiple joints—ranging from smaller joints such as fingers to larger joints including knees—and may include the hips and temporomandibular joints. This condition stems predominantly from pronounced tenosynovitis, with infrequent occurrences of erosions but frequent presentations of joint malformations such as camptodactyly and carpal dysplasia. Dermatological symptoms often emerge as the initial and second most common manifestation, typically presenting as non-pruritic erythematous papules during early childhood. Diagnostic histological examinations of skin biopsies reveal non-caseating granulomas. Ocular manifestations commonly include various forms of uveitis, which can significantly impair visual acuity and are predominantly bilateral [11,12]. Other ocular complications may include multifocal choroiditis and glaucoma. Despite intensive treatments, a considerable proportion of patients continue to experience active uveitis upon follow-up. Moreover, BS may also impact other bodily systems, manifesting in recurrent fevers and affecting cardiovascular, neurological, gastrointestinal, hepatic, and renal functions. Due to the rarity of BS and the absence of standardized therapeutic protocols, treatment strategies are generally based on expert consensus and tailored to individual cases [10].

Among the various challenges posed by BS, ocular involvement emerges as a particularly severe, degenerative, and often inadequately controlled issue [12,13]. The impact of ocular complications significantly contributes to the morbidity associated with BS, making it imperative to delve into the underlying molecular intricacies of ocular inflammation in this syndrome. The lack of predictive markers for disease progression at the ocular level poses a significant hurdle in the effective management of BS. This absence not only complicates the early identification of ocular involvement but also hinders the development of precise treatment strategies tailored to the unique characteristics of inflammation in BS patients [14]. In general, high-dose corticosteroids serve as initial therapy, with methotrexate recommended for joint or eye symptoms. Addition of an anti-TNFα is advised for persistent uveitis or arthritis. If symptoms persist, switching to another anti-TNFα is optimal. Non-responders to initial biologic therapy should switch to anti-interleukin 1, anti-interleukin 6, or tofacitinib [15]. The rarity of cases complicates the study of this pathology. At the Rheumatology Unit of the University of Padua, the sole Italian family diagnosed with BS carrying the p.E383K mutation in the *NOD2* gene has been under treatment for several years [16].

Hence, the primary objective of our study is to undertake a comprehensive investigation into the molecular aspects underlying BS-associated ocular inflammation. By unraveling the intricate molecular mechanisms at play in the ocular tissues affected by BS, we aim to identify potential biomarkers that could serve as predictive indicators of disease progression and response to treatment, specifically at the ocular level. This approach not only promises early detection of ocular involvement but also paves the way for the development of targeted therapeutic interventions to mitigate the severity of ocular manifestations in BS patients.

## 2. Results

### 2.1. Ocular Manifestations

**Figure 1:**
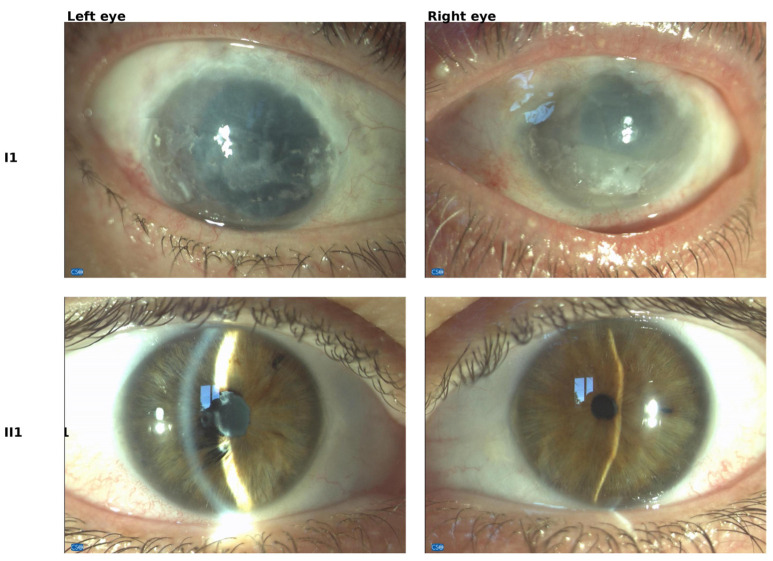
Ophthalmological evaluation of both left and right eyes of BS patients I1 and II1 with abnormal features.

The panel of images in Figure 1 summarizes the ophthalmological evaluation of BS patients with abnormal features. Of particular interest are the data for patient I1, who reported significant visual impairment during the examination. Visual acuity in the right eye was reduced to 1/10, while the evaluation of the left eye was inconclusive. The right eye exhibited aphakia due to a previous surgical intervention and corneal decompensation with a calcific band on the right side. This corneal manifestation resulted from chronic uveitis, characteristic of advanced stages in BS, correlating with the observed decline in visual acuity. The left eye showed sequelae of chronic inflammation with structural atrophy. Fundus oculi examination disclosed multiple peripheral retinal scars, indicative of past inflammatory episodes.

In patient II1, the right eye’s cornea appeared transparent, with some long-standing keratic precipitates, and a normal-depth and stable anterior chamber. There was sectoral iridal atrophy and peri-pupillary adhesions, along with 360-degree iridolenticular adhesions and annular fibrosis at the lens capsule. Additionally, remnants of a patent iridotomy were present. The fundus examination revealed normal findings. The left eye also presented a transparent cornea with some long-standing endothelial precipitates. The anterior chamber was of normal depth and quiet, with a miotic pupil and iridolenticular synechiae. The fundus examination showed normal findings.

Patients III1 and III2 exhibited transparent corneas with normal-depth and quiet anterior chambers, showing no signs of inflammation. The visual acuity of these patients, regularly monitored, remained stable at 10/10 in both eyes. The fundus examination revealed normal findings.

Healthy familial subjects and healthy controls also presented stable visual acuity at approximately 10/10 in both eyes.

### 2.2. Tears Proteomic Profiles

LC–MS/MS analyses were performed in technical triplicate, i.e., each sample was analyzed three times under the same instrumental and chromatographic conditions. A high correlation was found between the signal intensities obtained from the duplicated measurements of each pool, thus showing that results were technically sound and highly reproducible. Using this approach, a total of 387 proteins were identified, whose fold changes and statistical significance are reported in Table S1. Proteins were considered differentially expressed between pooled groups, i.e., (A) p.E383K carriers (n = 4), (B) healthy familial subjects (n = 3), and (C) healthy controls (n = 4), when the ratio between absolute intensity of the proteins was above 2 (overexpressed proteins in patients) or below −2 (under-expressed proteins in patients).

**Figure 2:**
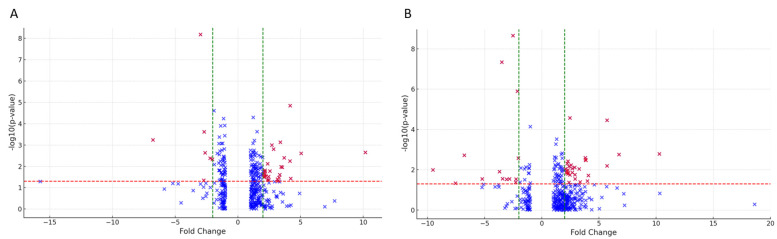
Volcano plots of differentially abundant proteins between BS-affected patients and unaffected familial subjects (plot **A**) or BS-patients and healthy controls (plot **B**). Positive values (on the right of each plot) on x-axis indicate higher expression, while negative values on the left suggest decreased expression. Significant data points above the *p*-value threshold (red dotted line) are considered statistically significant and highlighted in red. The green dotted lines bound the minimal fold-change for the most-differentially-expressed genes.

Differentially expressed proteins arise from comparisons between conditions A and B, and A and C, and are depicted in the volcano plots of Figure 2; significant findings are documented in Table S2. From the initial comparison, 27 proteins were significantly upregulated in the affected side of the family, while only seven were downregulated. Conversely, 30 proteins were significantly overexpressed in BS patients compared to healthy controls, with 14 being downregulated. Upon applying a more stringent significance criterion (fold change > |5|), three proteins were differentially expressed between BS subjects and other family members, while nine differed between BS patients and healthy controls (Table 1).

Among these, alpha-2-macroglobulin and immunoglobulin heavy constant gamma 4 were both overexpressed in BS patients compared to family members or healthy subjects.

To ensure that the differentially expressed proteins identified are associated with the disease, a comparison was performed between the two control groups (healthy and familial subjects) (Table S3) as an additional control for the specificity of the results. Overall, no significant differences were observed between the two control groups regarding the proteins differentially expressed in BS patients. Significant differences were detected in metabolism-related proteins, implying that these proteins are probably unique to each individual.

**Figure 3:**
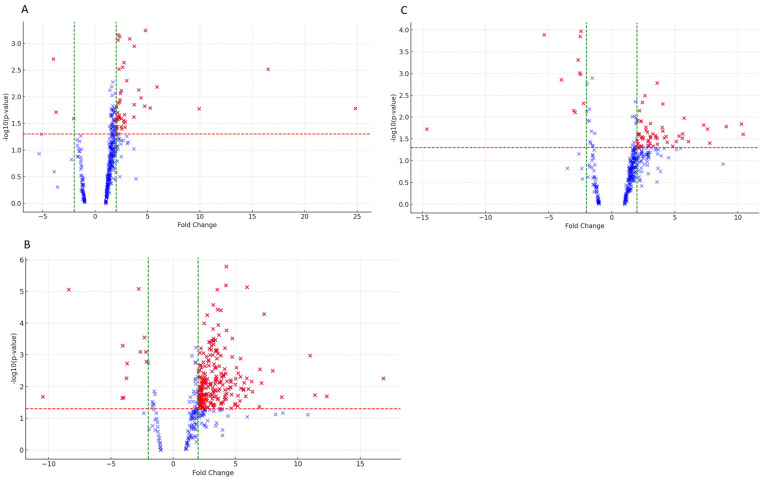
Volcano plots of differentially abundant proteins between I1 and II1 (plot **A**), I1 and III1-2 (plot **C**), and II1 and III1-2 (plot **B**). Positive values (on the right of each plot) on x-axis indicate higher expression, while negative values on the left suggest decreased expression. Significant data points above the *p*-value threshold (red dotted line) are considered statistically significant and highlighted in red. The green dotted lines bound the minimal fold change for the most-differentially-expressed genes.

To explore whether certain proteins play a significant role in the progression of the ocular degeneration observed during ophthalmological examinations, protein expression differences among BS-affected subjects were analyzed. We recall that, in order of severity, subject I1 exhibits the most severe ocular manifestations, followed by II1, with III1 and III2 displaying equivalent ocular status. We then opted to pool subjects III1 and III2 (referred to as III1-2) due to their similar clinical features. Differentially expressed proteins resulting from comparisons between I1 and II1, I1 and III1-2, and II1 and III1-2 are displayed in the volcano plots of Figure 3, and significant findings are reported in Table S4. The comparison between I1 and II1 revealed 47 significantly upregulated proteins and only three downregulated; in contrast, 217 proteins were significantly more expressed in I1 compared to III1-2 with 12 downregulated. II1 showed 58 proteins more expressed compared to III1-2 and 11 downregulated. Increasing the fold change threshold (FC > |5|), proteins differentially expressed among the affected subjects are detailed in Table 2.

To confirm the differentially expressed proteins identified in I1, the analysis was also performed on a second sample taken one year later. Overall, what was observed in the first analysis is reflected in the new sample. The differentially expressed proteins with FC > |5| in the first comparative analysis between I1 and II1 and between I1 and III1-2 were identified with similar values using the more recent sample (Table S5). Additionally, no significant differences in protein expression were detected between the old I1 sample and the new one, except for several keratins whose presence is intrinsically linked to sample processing (Table S6).

A comprehensive analysis using STRING network analysis (STRING; version 11.5) and/or DAVID software (version v2024q1) was conducted on significantly differentially expressed proteins with a fold change > |2| observed within and across BS family groups. Table 3 enumerates the gene ontology (GO) terms—biological processes (BP), cellular components (CC), and molecular functions (MF)—associated with over 10% of the proteins over-represented and under-represented in pooled BS patients relative to healthy controls and familial subjects, respectively. Acute-phase response, extracellular exosome, and protein binding were identified as the predominant GO-BP, -CC, and -MF. A similar analysis targeting BS-affected individuals, namely I1, II1, and the pooled III1-2 group, is presented in Table 4, highlighting extracellular exosome and protein binding as the main GO-CC and -MF.

**Figure 4:**
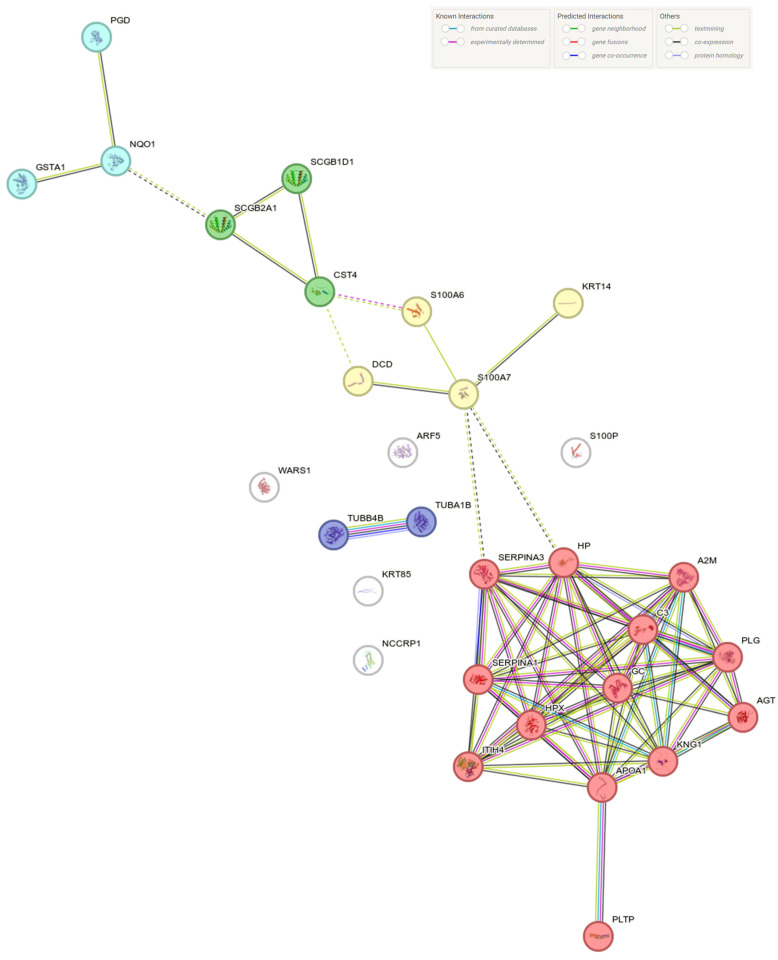
STRING network analysis of the proteins identified as significantly different between BS-affected patients and unaffected familial subjects. Lines connecting the different nodes represent functional and/or physical interactions.

**Figure 5:**
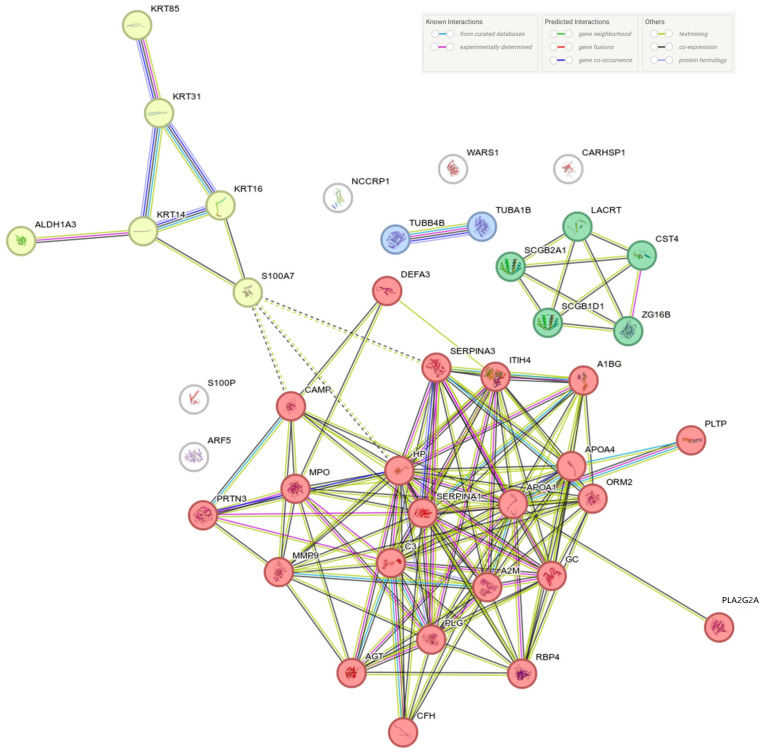
STRING network analysis of the proteins identified as significantly different between BS-affected patients and healthy controls. Lines connecting the different nodes represent functional and/or physical interactions.

STRING analysis of proteins differentially expressed in pooled BS patients versus healthy controls and familial subjects revealed distributions into four and five MCL clusters, respectively. Notably, in both instances, the clusters containing the highest number of proteins were those functionally assigned to stimulus response and the S100 protein family (Figure 4 and Figure 5). The large number of differentially expressed proteins in BS-affected individuals (I1, II1, and pooled III1-2) precluded the detailed cluster analysis in STRING.

## 3. Discussion

Ocular complications significantly enhance morbidity in Blau syndrome, underscoring the urgency of investigating the molecular details of ocular inflammation [12,13]. The lack of predictive markers for ocular disease progression complicates early detection and tailored treatment development for these patients. Tear fluid was chosen as the sample material because it is more accessible and less complex than other body fluids (e.g., plasma or serum) and its sampling is much less invasive [17]. As tear fluids represent the local milieu of the eye’s surface, we also thought that they would be a better source for investigating ocular surface pathologies. Moreover, ophthalmological evaluation did not reveal any ongoing inflammation in either of the two patients, both of whom have a quiet anterior chamber. In patient I1, the observed band keratopathy is the result of calcium salt accumulation at the corneal level, secondary to the characteristic chronic uveitis that distinguishes the more advanced stages of BS. This leads to superficial opacity at the Bowman membrane level, causing visual impairment and subsequent decrease in visual acuity. Fundus examination also revealed numerous peripheral retinal scars.

Molecular analysis, conducted through advanced mass spectrometry techniques, enabled us to identify proteins in the tear fluid differentially expressed in BS patients compared to both healthy and non-affected familial subjects. This in-depth analysis allows us to pinpoint specific proteins and possible pathways implicated in the ocular inflammation characteristic of BS, providing a molecular signature unique to the ocular involvement in this syndrome. Differential expression of keratins was observed across the various groups analyzed. However, keratins cannot be dismissed as potential contaminants during both the pre-analytical phase (sample collection) and the analytical phase (sample processing). Consequently, we have decided not to regard them as significant in our findings.

The results obtained from this spectrometric analysis offer several insights and partially confirm the pathogenetic autoinflammatory mechanisms already recognized in Blau syndrome at the systemic level [7,14]. This differential protein expression analysis, however, cannot differentiate between causal relationships, consequences, or mere correlations between protein expression and phenotypes. The expression of most individual proteins contributes minimally to the phenotype, although their cumulative effect can be significant. BS, however, imposes a significant burden on the organism, leading to substantial alterations in the proteome, including in ocular tissues. In light of these considerations, it is expected that the correlation between protein expression levels and a complex trait reflects disease status rather than a direct expression-to-trait link.

Among the most altered proteins in BS-affected patients are various molecules involved in innate immunity and infection defense. Notable among these are alpha-2 macroglobulin (A2M) and immunoglobulin heavy constant gamma 4 (IGHG4), which are highly expressed in BS carriers compared to either healthy or familial controls. A2M is a tetrameric protein involved in various extracellular processes dependent on proteolysis and is linked to innate immunity, capable of binding a wide array of molecules such as cytokines, growth factors, and apolipoproteins [18]. Additionally, α2M has the ability to stabilize misfolded proteins, thereby preventing their aberrant accumulation [19]. Another discovered function pertains to the modification α2M undergoes following interaction with hypochlorite (OCl-), an oxidizing agent produced by monocytes and neutrophils during an ongoing inflammatory process. While OCl- primarily serves an antimicrobial role, it also causes tissue damage and can alter protein structures, leading to their extracellular accumulation and predisposing to diseases such as Alzheimer’s, multiple sclerosis, and rheumatoid arthritis [20]. IGHG4 is a constant region of immunoglobulin heavy chain predicted to be involved in several processes, including activation of immune response, defense response to other organisms, and phagocytosis [21]. Located in blood microparticles and extracellular exosomes, its role in the tear fluid of BS patients could have potential implications in immune response. IgG4-related disease, a multi-organ fibro-inflammatory disease, is known for its characteristic histopathology showing lymphoplasmacytic infiltration and increased IgG4+ plasma cells [22]. The lacrimal gland is the most common ocular site of involvement. Scleritis and intraocular involvement in IgG4-related ophthalmic disease should be considered in any patient with multisystem inflammatory disease. This aspect related to immunoglobulins is a new area to explore in BS patients.

Additionally, some proteins are strongly downregulated in the BS patient pool compared to healthy controls, such as MPO and DEFA3, both part of the host defense system. Defensin-3 (DEFA3) is a protein produced by neutrophils with proven antibiotic, antifungal, and antiviral properties. DEFA3 belongs to the α-defensin family, stimulating macrophage phagocytosis [23]. α-defensins 1–3 stimulate TNF-α and IL-1 production, inhibiting IL-10. Myeloperoxidase (MPO) is a heme protein synthesized during myeloid differentiation that constitutes the major component of neutrophil azurophilic granules. MPO catalyzes the formation of various reactive oxygen species that contribute to inflammatory tissue damage [24].

However, a decreased level in the BS pool could be misleading, because BS subjects within the BS pool had different degrees of disease severity. Indeed, MPO and DEFA3 are overexpressed in I1, the older and more severely affected subject, compared to other p.E383K carriers. This aligns with the literature stating that MPO levels usually correlate with disease severity [24]. Further confirmation comes from the analysis performed one year later, where the new sample from patient I1 exhibits overexpression of MPO and DEFA3. Although based on preliminary data, the progression of the disease could be a contributing factor to the differential expression of these proteins.

The augmented concentrations of MPO and DEFA3 are also concomitant with elevated levels of azurocidin (AZU1) in the same subject I1 compared to other p.E383K carriers. AZU1 is a chemotactic antibacterial glycoprotein specific to monocytes and fibroblasts derived from neutrophil granules. Its cytotoxic action is limited to many species of Gram-negative bacteria. Azurocidin is rapidly mobilized by migrating polymorphonuclear leukocytes (PMNs). Initially recognized for its antimicrobial effects [25], Azurocidin is now understood to act as an alarm for the immune system, serving as a crucial mediator during the initiation of the immune response [26]. Azurocidin released by PMN secretory vesicles or primary granules acts as a chemoattractant and activator of monocytes and macrophages [27]. Its release is implicated in the expression of adhesion molecules on the extracellular matrix surface, contributing to increased leukocyte adhesion. The release of granule content is tightly regulated; however, it is known that their unregulated release can aggravate tissue damage and could be detrimental to the host [28]. Targeting azurocidin release may have therapeutic potential in inflammatory conditions. Given that AZU1 expression levels are known to be positively associated with the severity of different diseases, it could be a good candidate for identifying the potential for more severe disease progression. Numerous findings in the literature suggest varied roles of neutrophils on the ocular surface [28], such as infiltration, degranulation, neutrophil extracellular trap (NET) formation, and autophagy, involved in the pathogenesis of ocular surface diseases. The identification of differential expression of proteins related to neutrophils and neutrophil azurophilic granules underscores their potential clinical and molecular implications for BS-associated uveitis. Concerning uveitis, it is noteworthy that research specifically concentrating on the proteomics of tears in relation to uveitis is limited. Angeles-Han et al. [29] compared the proteomic profiles of tear samples between children with juvenile idiopathic arthritis-associated uveitis (JIA-U) and those with idiopathic uveitis. The study found elevated protein levels related to inflammatory arthritis in JIA-U, suggesting that tear protein expression differences could aid in distinguishing these conditions. Similarly, Eidet et al. [30] investigated proteomic variations in tear fluid between the affected and healthy ipsilateral eyes in cases of acute anterior uveitis (AAU), identifying detectable protein changes. Increased levels of serpin family A member 3 (SERPINA3) were observed in the tears of AAU cases, indicating an acute-phase response. This protein level is also augmented in our study of the BS-pooled group vs. healthy controls, as well as in the family members with ocular damages (subjects I1 and II1) vs. the other p.E383K carriers (III1-2). Given these findings, it is plausible to suggest that the majority of the differentially expressed proteins we identified are specific to BS-associated uveitis.

Another highly expressed protein in I1 compared to the other p.E383K carriers is aldehyde dehydrogenase (ALDH3A1), one of the most abundantly expressed proteins in the corneal epithelium, which constitutes 5–50% of the total soluble protein fraction in mammals [31]. As a member of the ALDH protein superfamily, it plays a critical and multifunctional role in corneal protection from UV-induced oxidative stress. Its roles include metabolizing toxic aldehydes, direct UV light absorption, antioxidant function, maintaining corneal properties, and reducing DNA synthesis and cell proliferation. ALDH3A1’s higher expression in I1 suggests adaptation to severe ocular involvement, protecting the cornea from external stress.

The identification of the same highly expressed proteins in I1 compared to other p.E383K carriers across two different tear samples strengthens the robustness and reproducibility of our findings. However, it would be appropriate to repeat the analysis of the whole family in a future experiment.

By identifying novel potential molecular markers associated with ocular inflammation, we would pave the way for the development of targeted therapies aimed at modulating these specific molecular pathways. This study possesses certain limitations, notably the constrained cohort size comprising solely familial cases of BS carrying the p.E383K variant in *NOD2*. An expansion of the cohort to include not only healthy individuals but also BS patients with alternative pathogenic variants and those with uveitis might facilitate validation of the differentially expressed proteins identified in this study as potential biomarkers of ocular severity in BS. To confirm our experimental results and expand the sample size, we will conduct forthcoming experiments involving a more extensive cohort of patients with *NOD2* variants and controls across multiple Italian centers. Additionally, diverse analytical methods, such as multiplex arrays on tear fluids, will be employed to enhance the robustness and reproducibility of our findings. These will also validate our initial observations, ensuring the reliability of the conclusions drawn. Additionally, the acknowledged inter-day variability in tear fluid composition [32] warrants further investigation through the collection of multiple samples. Furthermore, there are other known potential confounding factors such as age, sex, diet, and pharmacological treatment, which may influence the results. Given the small sample size, it is currently not possible to calculate the impact of these variables; however, this will be the focus of forthcoming experiments involving a more extensive cohort of subjects. Nevertheless, biological confounding factors will always be present. However, our proteomic methods have been designed to minimize the effects of technological confounding factors by employing a standardized procedure.

In conclusion, proteomics, along with heightened scientific and clinical interest in non-invasive patient sampling methods, has made the field of tear fluid analysis increasingly attractive for disease diagnosis and monitoring. This approach has the potential not only to improve treatment precision but also to provide insights into novel therapeutic targets, thereby advancing the management of BS-related ocular complications.

## 4. Material and Methods

### 4.1. Patients

**Figure 6:**
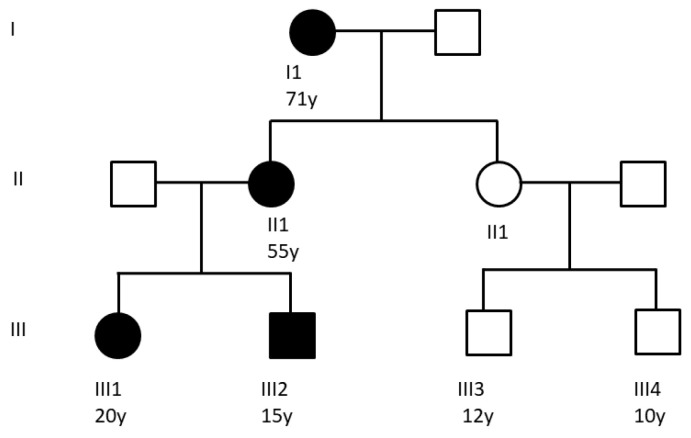
Representation of the familial transmission of BS in the patient group considered in the study. The p.E383K mutation in the NACHT domain of *NOD2* gene is present in patients I1, II1, III1, and III2, confirmed by genetic analysis. Family tree modified from [2].

Seven subjects belonging to the same family (Figure 6), 4 of which were carriers of a BS-associated mutation in *NOD2* (p.E383K), already characterized in [2], were recruited together with 4 healthy controls. Subject I1 were reassessed after one year by collecting new tear fluid samples. Subject II2, along with her children III3 and III4, did not show signs or symptoms related to the pathology, consistent with negative genetic tests for the p.E383K mutation in *NOD2*. The disease symptoms, for those affected by BS, were reported under control at the time of enrolment. The study was approved by the Local Ethics Committee of the University Hospital of Padua, and all subjects gave their full informed written consent at enrolment.

#### Clinical Features of NOD2 p.E383K Carriers

The p.E383K carriers (I1, II1, III1, and III2) were monitored at the Rheumatology Unit, Department of Medicine of the University Hospital of Padua since the onset of symptoms. Patient I1, a 71-year-old Caucasian woman, was first examined at the age of 31 in 1984. She presented with a clinical history characterized by papulo-nodular eruption, polyarthritis in hands and feet, chronic bilateral uveitis, and glaucoma, with subsequent cataract development. Over the years, the ocular involvement necessitated iridectomy at the age of 24 and later cataract surgery at the age of 31. The patient reported clinical onset during puberty, specifically with cutaneous manifestations and symmetric arthritis in hands, feet, and wrists, both characterized by periods of exacerbation controlled with NSAIDs and periods of remission. At the initial visit, the patient exhibited bilateral arthritis in metacarpophalangeal and proximal interphalangeal joints, as well as involvement similar to the first and second metatarsophalangeal joints. The affected joints were slightly swollen but not red or painful, and there was no camptodactyly or significant radiological abnormalities, except for slight periosteal thickening. On the back of the hands and feet and on the extensor surfaces of the legs, the patient had widespread asymptomatic brown papules with subcutaneous nodules between 5 and 30 mm in diameter. Histological examination of the skin lesions revealed non-caseating granulomas with histiocytes and multinucleated giant cells. Laboratory investigations did not show any abnormal findings, particularly no elevation of inflammatory markers. The definitive diagnosis came in 2005 with genetic testing revealing a p.E383K mutation in the NACHT domain of *NOD2*. This mutation, a novel genetic alteration associated with BS, was later found in other patients affected by the syndrome. The patient has been undergoing treatment with the anti-TNF-alpha biological drug Adalimumab (Humira 40 mg/14 days), which has provided good control of cutaneous and ocular manifestations. Among the two daughters of patient I1, only one (II1) inherited the characteristic mutation in NOD2, passing it on to her children, III1 and III2, aged 14 and 9 years, respectively. Patient II1, in follow-up since the age of 5, exhibited similar, although less severe, cutaneous manifestations, uveitis, and arthritis in the fingers attributable to BS. Her clinical state at the time of this study was good, while she was receiving methylprednisolone 4 mg/day. Patients III1 and III2 carried the p.E383K mutation inherited from patient II1 (Figure S1). Although clinical symptoms in these descendants are currently subtle, patient III1 has started to show cutaneous and articular symptoms compatible with the onset of the disease.

### 4.2. Tears Sampling

All subjects underwent ophthalmological examination at the Ophthalmology Clinic of the University Hospital of Padua. BS-affected subjects underwent a visual acuity evaluation, optical coherence tomography (OCT) to acquire cross-sectional images of the tissue structure, and tears collection. All healthy subjects, instead, underwent a visual acuity evaluation and the tears sampling.

Ophthalmologists collected tear samples from all subjects using Schirmer strips which are routinely used for dry eye evaluation, without adding any anesthetics. A Schirmer strip was placed into the temporal inferior fornix of each eye (approximately 6 mm from the lateral canthus), avoiding the corneal surface, and removed when fully saturated (maximum 5 min). Each Schirmer strip was placed in a microcentrifuge tube on ice and stored at −80 °C until processing.

### 4.3. Protein Extraction

Each strip was soaked for 15 min on ice in 500 μL of urea lysis buffer (8 M urea, 100 mM NaH_2_PO_4_, pH 8.5), including 5 μL (100× stock) HALT protease and phosphatase inhibitor cocktail (Pierce, Appleton, WI, USA). After vortexing and centrifuging 15,000× *g* for 5 min at 4 °C, protein supernatants were quantified using BCA assay (Thermofisher, Waltham, MA, USA). An average protein concentration of approx. 1 µg/µL (mean ± S = 1.31 ± 0.44) was detected. Then, 14 µg of each sample was loaded onto a precast gel (NuPAGE, 4–12% Bis-Tris, Life Technologies, Monza, Italy) for protein qualitative evaluation via SDS-PAGE gel electrophoresis.

### 4.4. Protein Digestion

Next, 40 µL of each sample was diluted in 200 µL with the washing buffer (WB, Urea 8 M, Tris-HCl 100 mM, pH 8.5) and loaded into a Vivacon 500 filter (Sartorius, Göttingen, Germany) to perform a FASP (filter-aided sample preparation) protocol for protein digestion. Three washes with WB and subsequent centrifugation at 18,000× *g* were performed. Proteins retained in the filter were reduced with 50 mM dithiothreitol (Fluka) in WB (incubation at 55 °C for 30 min) and alkylated with 50 mM iodoacetamide (Sigma-Aldrich, Milan, Italy) in WB (incubation 20 min at room temperature and in the dark). Proteins were washed twice with WB, once with NH_4_HCO_3_ 100 mM, and once with NH_4_HCO_3_ 50 mM, as previously described [33]. Protein digestion was performed overnight at 37 °C, adding 0.8 µg of trypsin (Promega, Madison, WI, USA). Three extraction steps, performed by washing with 50 mM NH_4_HCO_3_ and centrifuging at 18,000× *g* for 10 min, allowed for collection of tryptic peptides that were finally acidified to pH 3 with formic acid 0.1% to a final volume of 1 mL. Untargeted proteomic studies described below were performed not on eye samples but pooling left and right eye samples for each patient. Prior to further analysis, pooled samples were desalted with C18 cartridges (Sep-Pak, Waters, Milford, MA, USA), dried under vacuum, and stored at −20 °C.

### 4.5. Mass Spectrometry and Protein Identification

Untargeted proteomic analyses were performed by LC–MS/MS with an LTQ-Orbitrap XL mass spectrometer (Thermo Fisher Scientific, Waltham, MA, USA) coupled with a nano-HPLC Ultimate 3000 (Dionex–Thermo Fisher Scientific, Waltham, MA, USA). Peptides were separated in a 10 cm pico-frit column (75 μm I.D., New Objectives), packed in-house with C18 material (Aeris Peptide 3.6 μm XB-C18, Phenomenex, Torrance, CA, USA), using a linear gradient from 3 to 50% ACN/0.1 FA in 90 min at a flow rate of 250 nL/min. Three technical replicates were acquired for each sample.

Raw data were analyzed with the Proteome Discoverer software (version 1.4, Thermo Fisher Scientific) interfaced to a Mascot server (version 2.2.4, Matrix Science, London, UK) and searched against the human section of the Uniprot Database (www.uniprot.org, version 20150401, 90411 sequences, accessed on 1 June 2022) using trypsin as digesting enzyme. The software calculated intensities of each identified protein that were used as ratios against control samples, which include both familial subjects and healthy individuals. Utilizing this technique, the software-calculated protein intensities revealed significant differences in protein expression levels between BS patients and combined groups of p.E383K carriers, healthy familial subjects, and healthy controls. False discovery rate (FDR) was set at 0.01. Only proteins identified with at least 2 peptides were considered.

### 4.6. Statistical and Bioinformatic Analysis

Descriptive statistics were used to explore data by using mean and standard deviation, where appropriate. The Student’s *t*-test was employed for identifying differentially expressed proteins. Benjamini–Hochberg (BH) correction was performed for multiple comparisons, and corrected *p*-values less than 0.05 were considered significant. Volcano plots were used to visualize differential gene expression patterns using R software version 4.3.2 (R Foundation for Statistical Computing, Vienna, Austria), specifically utilizing the “ggplot” function. To highlight potential functional interactions between proteins identified by the untargeted proteomic analyses and enriched pathways and biological functions, the tools STRING version 11.5 [34] and DAVID Bioinformatic Resources 6.8 v2024q1 [35] were used.

## Figures and Tables

**Table 1:** Significantly differentially abundant proteins between BS-affected patients (A; n = 4) and unaffected familial subjects (B; n = 3) or healthy controls (C; n = 4) groups. Only proteins with a fold change (FC) ≥ 5 or ≤−5 are reported. * Benjamini–Hochberg correction for multiple comparisons.

**A vs. B**			
**Gene Name**	**Protein Description**	**Fold Change**	*p* Value *
*S100A7*	S100 calcium binding protein A7	−6.78	0.0006
*A2M*	Alpha-2 macroglobulin	5.03	0.0025
*IGHG4*	Immunoglobulin heavy constant gamma 4	10.17	0.0022
**A vs. C**			
**Gene Name**	**Protein Description**	**Fold Change**	*p* Value *
*CAMP*	Cathelicidin antimicrobial peptide	−9.53	0.0050
*PRTN3*	Proteinase 3	−7.56	0.0260
*DEFA3*	Defensin alpha 3	−6.78	0.0010
*MPO*	Myeloperoxidase	−5.21	0.0218
*SERPINA3*	Serpin family A member 3	5.705312	0.0030
*HP*	Haptoglobin	5.706	2.47 × 10^−5^
*A2M*	Alpha-2 macroglobulin	6.746384	0.0007
*IGHG4*	Immunoglobulin heavy constant gamma 4	10.27741	0.0010
*KRT31*	Keratin 31	60.95701	0.0190

**Table 2:** Significantly differentially abundant proteins between I1 and II1, I1 and III1-2, and II2 and III1-2. Only proteins with a fold change (FC) ≥ 5 or ≤−5 are reported. * Benjamini–Hochberg correction for multiple comparisons.

**I1 vs. II1**			
**Gene Name**	**Protein Description**	**Fold Change**	***p*** Value *
*KRT4*	Keratin 4	5.25	0.0162
*ALDH3A1*	Aldehyde dehydrogenase 3 family member A1	5.90	0.0065
*DEFA3*	Defensin alpha 3	9.93	0.0168
*MPO*	Myeloperoxidase	16.50	0.0030
*AZU1*	Azurocidin	24.84	0.0164
**I1 vs. III1-2**			
**Gene Name**	**Protein Description**	**Fold Change**	***p*** Value *
*PRR27*	Proline-rich 27	−10.47	0.0209
*IGLV3-21*	Immunoglobulin lambda variable 3–21	−8.39	8.76 × 10^−6^
*ALDH1A1*	Aldehyde dehydrogenase 1 family member A1	5.00	0.0055
*APOA4*	Apolipoprotein A4	5.05	0.0024
*AKR1C1*	Aldo-keto reductase family 1 member C1	5.17	0.0406
*ITIH2*	Inter-alpha-trypsin inhibitor heavy chain 2	5.18	0.0192
*CRYZ*	Crystallin zeta	5.20	0.0037
*AK1*	Adenylate kinase 1	5.35	0.0283
*AKR1C2*	Aldo-keto reductase family 1 member C2	5.39	0.0049
*MYH14*	Myosin heavy chain 14	5.40	0.0013
*CRYAB*	Crystallin alpha B	5.42	0.0113
*TALDO1*	Transaldolase 1	5.60	0.0119
*DEFA3*	Defensin alpha 3	5.64	0.0127
*KRT4*	Keratin 4	5.69	0.0075
*KRT13*	Keratin 13	5.81	0.0201
*NQO1*	NAD(P)H quinone dehydrogenase 1	5.89	0.0054
*GNB2*	G protein subunit beta 2	5.92	7.29 × 10^−6^
*LCN2*	Lipocalin 2	6.03	0.0120
*F2*	Coagulation factor II, thrombin	6.28	0.0068
*SERPINA3*	Serpin family A member 3	6.35	0.0145
*HPX*	Hemopexin	6.90	0.0434
*MPO*	Myeloperoxidase	6.94	0.0028
*AZU1*	Azurocidin	7.07	0.0076
*KRT7*	Keratin 7	7.28	5.16 × 10^−5^
*AGT*	Angiotensinogen	7.97	0.0031
*GLUL*	Glutamate–ammonia ligase	8.70	0.0213
*SPARCL1*	SPARC-like 1	10.98	0.0010
*APOC3*	Apolipoprotein C3	11.36	0.0184
*SERPING1*	Serpin family G member 1	12.32	0.0201
*ALDH3A1*	Aldehyde dehydrogenase 3 family member A1	16.87	0.0055
**II1 vs. III1-2**			
**Gene Name**	**Protein Description**	**Fold Change**	***p*** Value *
*PRR27*	Proline-rich 27	−14.66	0.0189
*CXCL17*	C–X–C motif chemokine ligand 17	−5.36	0.0001
*ITIH1*	Inter-alpha-trypsin inhibitor heavy chain 1	5.10	0.0364
*SERPINA3*	Serpin family A member 3	5.32	0.0272
*AHSG*	Alpha 2-HS glycoprotein	5.61	0.0303
*IGHG2*	Immunoglobulin heavy constant gamma 2	5.62	0.0242
*KNG1*	Kininogen 1	5.73	0.0104
*SERPINB1*	Serpin family B member 1	6.09	0.0360
*GLUL*	Glutamate–ammonia ligase	7.30	0.0151
*F2*	Coagulation factor II, thrombin	7.59	0.0186
*HPX*	Hemopexin	7.77	0.0391
*VTN*	Vitronectin	9.08	0.0163
*AGT*	Angiotensinogen	10.29	0.0143
*SERPING1*	Serpin family G member 1	10.41	0.0248
*KRT83*	Keratin 83	78.90	0.0420
*KRT33B*	Keratin 33B	192.48	0.0294

**Table 3:** Enrichment analysis of GO terms associated with the proteins identified as differently abundant in BS-affected patients (A; n = 4) vs. unaffected familial subjects (B; n = 3) and (A) vs. healthy controls (C; n = 4). The top most significant GO terms, with more than 10% of genes associated, are listed. Significant *p*-values Benjamini–Hochberg-corrected for multiple comparisons in bold.

**A vs. B**			
**Category**	**Term**	**%**	***p*** **Value**
GOTERM_CC_DIRECT	GO:0070062~extracellular exosome	81.82	**1 × 10^−26^**
GOTERM_MF_DIRECT	GO:0005515~protein binding	79.55	**0.02**
GOTERM_CC_DIRECT	GO:0005615~extracellular space	75.00	**2.23 × 10^−24^**
GOTERM_CC_DIRECT	GO:0005576~extracellular region	70.45	**3.99 × 10^−20^**
GOTERM_CC_DIRECT	GO:0072562~blood microparticle	38.64	**4.62 × 10^−24^**
GOTERM_CC_DIRECT	GO:0035578~azurophil granule lumen	18.18	**7.55 × 10^−10^**
GOTERM_BP_DIRECT	GO:0006953~acute-phase response	13.64	**2.12 × 10** **^−8^**
GOTERM_BP_DIRECT	GO:0019731~antibacterial humoral response	13.64	**2.98 × 10^−7^**
GOTERM_CC_DIRECT	GO:0031093~platelet alpha granule lumen	13.64	**3 × 10^−7^**
GOTERM_MF_DIRECT	GO:0042803~protein homodimerization activity	13.64	**0.02**
GOTERM_BP_DIRECT	GO:0006958~complement activation, classical pathway	11.36	**2.26 × 10^−6^**
GOTERM_BP_DIRECT	GO:0042742~defense response to bacterium	11.36	**4.30 × 10^−4^**
GOTERM_BP_DIRECT	GO:0006508~proteolysis	11.36	**0.04**
GOTERM_CC_DIRECT	GO:0034774~secretory granule lumen	11.36	**1.01 × 10^−4^**
GOTERM_MF_DIRECT	GO:0004867~serine-type endopeptidase inhibitor activity	11.36	**8.71 × 10^−5^**
GOTERM_MF_DIRECT	GO:0005200~structural constituent of cytoskeleton	11.36	**9.35 × 10^−5^**
GOTERM_MF_DIRECT	GO:0005102~receptor binding	11.36	**0.01**
**A vs. C**			
**Category**	**Term**	**%**	***p*** **Value**
GOTERM_MF_DIRECT	GO:0005515~protein binding	82.35	**0.05**
GOTERM_CC_DIRECT	GO:0070062~extracellular exosome	79.41	**1.66 × 10^−19^**
GOTERM_CC_DIRECT	GO:0005576~extracellular region	64.71	**3.02 × 10^−13^**
GOTERM_CC_DIRECT	GO:0005615~extracellular space	61.76	**4.64 × 10^−13^**
GOTERM_CC_DIRECT	GO:0072562~blood microparticle	44.12	**3.59 × 10^−22^**
GOTERM_BP_DIRECT	GO:0006953~acute-phase response	14.71	**5.50 × 10^−7^**
GOTERM_BP_DIRECT	GO:0006958~complement activation, classical pathway	14.71	**8.13 × 10^−7^**
GOTERM_CC_DIRECT	GO:0031093~platelet alpha granule lumen	14.71	**4.20 × 10^−6^**
GOTERM_MF_DIRECT	GO:0004867~serine-type endopeptidase inhibitor activity	14.71	**3.64 × 10^−5^**
GOTERM_MF_DIRECT	GO:0005102~receptor binding	14.71	**4.16 × 10^−3^**
GOTERM_BP_DIRECT	GO:0050853~B cell receptor signaling pathway	11.76	**1.27 × 10^−4^**
GOTERM_BP_DIRECT	GO:0019731~antibacterial humoral response	11.76	**1.86 × 10^−4^**
GOTERM_BP_DIRECT	GO:0002250~adaptive immune response	11.76	**0.04**
GOTERM_CC_DIRECT	GO:0042571~immunoglobulin complex, circulating	11.76	**3.16 × 10^−7^**
GOTERM_CC_DIRECT	GO:0071735~IgG immunoglobulin complex	11.76	**1.07 × 10^−6^**
GOTERM_CC_DIRECT	GO:0035578~azurophil granule lumen	11.76	**4.18 × 10^−4^**
GOTERM_CC_DIRECT	GO:0034774~secretory granule lumen	11.76	**8.49 × 10^−4^**
GOTERM_CC_DIRECT	GO:0005788~endoplasmic reticulum lumen	11.76	**0.01**
GOTERM_MF_DIRECT	GO:0034987~immunoglobulin receptor binding	11.76	**2.18 × 10^−6^**
GOTERM_MF_DIRECT	GO:0048306~calcium-dependent protein binding	11.76	**3.92 × 10^−4^**
GOTERM_MF_DIRECT	GO:0003823~antigen binding	11.76	**1.89 × 10^−3^**

**Table 4:** Enrichment analysis of GO terms associated with the proteins identified as differently abundant in the p.E383K carriers. The top most significant GO terms, with more than 10% of genes associated, are listed. Significant *p*-values Benjamini–Hochberg-corrected for multiple comparisons in bold.

**I1 vs. II1**			
**Category**	**Term**	**%**	***p*** **Value**
GOTERM_MF_DIRECT	GO:0005515~protein binding	85.71	**3.8 × 10^−3^**
GOTERM_CC_DIRECT	GO:0005829~cytosol	69.39	**1.86 × 10^−9^**
GOTERM_CC_DIRECT	GO:0070062~extracellular exosome	65.31	**7.76 × 10^−19^**
GOTERM_CC_DIRECT	GO:0005737~cytoplasm	44.9	**0.01**
GOTERM_CC_DIRECT	GO:0005634~nucleus	42.86	**0.04**
GOTERM_CC_DIRECT	GO:0005615~extracellular space	34.69	**4.31 × 10^−6^**
GOTERM_CC_DIRECT	GO:0005576~extracellular region	22.45	**0.03**
GOTERM_MF_DIRECT	GO:0042802~identical protein binding	20.41	**0.03**
**I1 vs. III1-2**			
**Category**	**Term**	**%**	***p*** **Value**
GOTERM_CC_DIRECT	GO:0070062~extracellular exosome	84.14	**3.53 × 10^−147^**
GOTERM_MF_DIRECT	GO:0005515~protein binding	81.06	**5 × 10^−7^**
GOTERM_CC_DIRECT	GO:0005829~cytosol	62.11	**8.42 × 10^−29^**
GOTERM_CC_DIRECT	GO:0005737~cytoplasm	52.42	**1.62 × 10^−15^**
GOTERM_CC_DIRECT	GO:0005576~extracellular region	48.02	**3.61 × 10^−46^**
GOTERM_CC_DIRECT	GO:0005615~extracellular space	42.29	**3.19 × 10^−39^**
GOTERM_CC_DIRECT	GO:0005634~nucleus	40.53	**2.25 × 10^−4^**
GOTERM_CC_DIRECT	GO:0005886~plasma membrane	33.92	**7.41 × 10^−3^**
GOTERM_MF_DIRECT	GO:0042802~identical protein binding	22.47	**2.08 × 10^−9^**
GOTERM_CC_DIRECT	GO:0072562~blood microparticle	19.82	**1.74 × 10^−51^**
GOTERM_MF_DIRECT	GO:0003723~RNA binding	16.74	**1 × 10^−5^**
GOTERM_MF_DIRECT	GO:0005509~calcium ion binding	14.10	**1.11 × 10^−9^**
GOTERM_CC_DIRECT	GO:0005925~focal adhesion	13.66	**6.46 × 10^−16^**
GOTERM_CC_DIRECT	GO:0034774~secretory granule lumen	12.78	**5.95 × 10^−30^**
GOTERM_CC_DIRECT	GO:0005788~endoplasmic reticulum lumen	11.45	**7.87 × 10^−15^**
GOTERM_CC_DIRECT	GO:0005739~mitochondrion	11.45	**0.01**
GOTERM_CC_DIRECT	GO:1904813~ficolin-1-rich granule lumen	10.57	**7.23 × 10^−22^**
GOTERM_BP_DIRECT	GO:0007165~signal transduction	10.13	**0.03**
GOTERM_CC_DIRECT	GO:0009986~cell surface	10.13	**2.62 × 10^−6^**
GOTERM_MF_DIRECT	GO:0045296~cadherin binding	10.13	**3.46 × 10^−11^**

## Data Availability

The raw data supporting the conclusions of this article will be made available by the authors on request.

## Supplementary Materials

The supporting information can be downloaded at: https://www.mdpi.com/article/10.3390/ijms25158387/s1.
