# Supplementary material for: Proteomic Profiling of Tears in Blau Syndrome Patients in Identification of Potential Disease Biomarkers
Source: Int J Mol Sci. 2024 Aug 1;25(15):8387. doi: 10.3390/ijms25158387 (PMC11312868; doi:10.3390/ijms25158387)
Supplement: Supplementary file 1 [file ijms-25-08387-s001.zip › Figure S1.pdf]

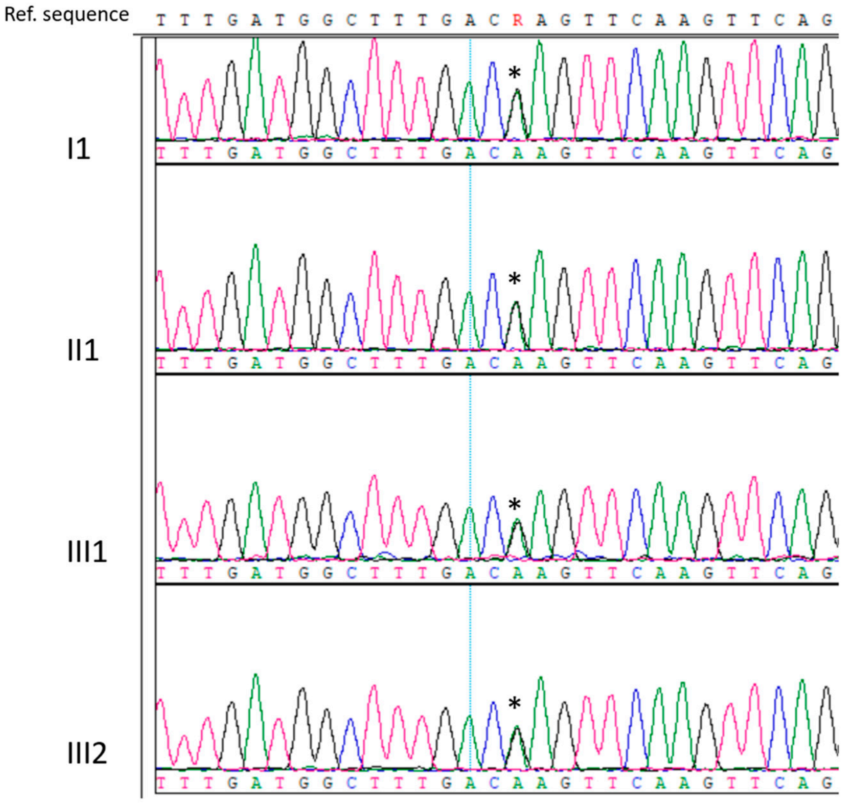

Supplementary Figure S1: Alignment of Sanger sequencing of the 4 family members (I1, II1, III1, III2) carrying the p.E383K mutation in *NOD2* gene (NM_022162.3). The asterisks (*) indicate the heterozygous mutation c.1147G>A (p.E383K).

Genomic DNA was extracted from blood samples of the enrolled family members and its concentration is determined with Nanodrop (ND 1000 Spectrophotomer). Exon 4 of *NOD2* was amplified using primers designed with PRIMER3 (primer and PCR condition available upon request). After purification (Exonuclease I and Shrimp Alkaline Phosphatase; EXO-SAP), 3ng of DNA were sequenced by ABI 3730XL DNA sequencer (Applied Biosystems). LASERGENE software package (SeqMan II, DNASTAR) was used to assemble the sequences (Supplementary Figure 1).
