# Supplementary material for: Proteomic Profiling of Tears in Blau Syndrome Patients in Identification of Potential Disease Biomarkers
Source: Int J Mol Sci. 2024 Aug 1;25(15):8387. doi: 10.3390/ijms25158387 (PMC11312868; doi:10.3390/ijms25158387)
Supplement: Supplementary file 1 [file ijms-25-08387-s001.zip › Table S3.pdf]

Supplementary Table S3: Differential expressed proteins in healthy familial subjects vs healthy controls

Protein	Protein Description	B/C	p-value
KRT13	Keratin 13	-11.62	3.69E-02
KRT4	Keratin 4	-7.79	2.85E-02
ARF5	ADP-ribosylation factor 5 (Fragment)	-7.20	8.75E-03
SELENBP1	Methanethiol oxidase	5.02	2.81E-05
AKR1C2	Aldo-keto reductase family 1 member C2	5.22	4.38E-02
ASS1	Argininosuccinate synthase	5.27	4.84E-03
AKR1C1	Aldo-keto reductase family 1 member C1	5.52	1.43E-02
SORD	Sorbitol dehydrogenase	9.03	7.03E-05
