# Supplementary material for: Proteomic Profiling of Tears in Blau Syndrome Patients in Identification of Potential Disease Biomarkers
Source: Int J Mol Sci. 2024 Aug 1;25(15):8387. doi: 10.3390/ijms25158387 (PMC11312868; doi:10.3390/ijms25158387)
Supplement: Supplementary file 1 [file ijms-25-08387-s001.zip › Table S5.pdf]

Table S5: Significant differentially expressed proteins in the second sample (1 year later) of I1 vs the other p.E383K-carriers (II1 and pooled III1 and III2). Asterisk (*) marks the differentially expressed proteins already identified in the I1 previous sample.

I1 NEW VS II1

GENE NAME	Protein description	fold change	p value
**TYMP**	Thymidine phosphorylase	-5.16	0.0499
**B2M***	beta 2 microglobulin	5.43	0.0028
**MB**	Myoglobin	6.01	0.0657
**ALDH3A1***	Aldehyde Dehydrogenase 3 Family Member A1	6.19	0.0064
**KRT16**	Keratin 16	8.13	0.0003
**DEFA3***	Defensin alpha 3	9.20	0.0077
**MPO***	Myeloperoxidase	9.80	0.0522
**AZU1***	Azurocidin	21.81	0.0000

I1 NEW VS POOL III1-2

GENE NAME	Protein description	fold change	p value
**PRR27***	Proline rich 27	-12.97	0.0196
**IGLV3-21***	Immunoglobulin lambda variable 3-21	-9.25	0.0001
**PLTP**	Phospholipid transfer protein	-5.35	0.0033
**SPRR1A**	Small prolin rich protein 1A	5.06	0.0008
**MYH14***	Myosin heavy chain 14	5.06	0.0010
**A2M**	alpha 2 microglobulin	5.16	0.0192
**MYL12A**	Myosin light chain 12A	5.17	0.0116
**AK1***	Adenylate kinase 1	5.21	0.0001
**DEFA3***	Defensin alpha 3	5.23	0.0026
**YWHAE**	Tyrosine 3-Monooxygenase/Tryptophan 5-Monooxygenase activation protein epsilon	5.47	0.0001
**TACSTD2**	Tumor associated calcium signal transducer 2	5.48	0.0194
**APOH**	Apolipoprotein H	5.57	0.0001
**AKR1C1***	Aldo-keto reductase family 1 member C1	5.77	0.0020
**MMP9***	Metalloproteinase 9	5.85	0.0045
**CRYZ***	Crystallin zeta	5.86	0.0055
**APOA1**	Apolipoprotein A1	6.12	0.0193
**AZU1***	Azurocidin	6.21	0.0030
**HRG**	Histidine rich glycoprotein	6.24	0.0016
**AMBP**	Alpha 1 microglobulin/bikunin precurson	6.34	0.0062
**HMGN2***	High mobility group nucleosomal binding domain 2	6.47	0.0044
**HPX***	Hemopexin	6.50	0.0123
**VTN**	Vitronectin	6.77	0.0085
**SERPINA3***	serpin family A member 3	6.90	0.0234
**F2***	coagulation factor II, thrombin	7.43	0.0235
**AGT***	Angiotensinogen	7.91	0.0051
**ITIH2***	Inter alpha trypsin inhibitor heavy chain 2	8.17	0.0101
**KRT16**	keratin 16	8.89	0.0004
**GLUL***	glutamate-ammonia ligase	11.07	0.0068
**SHMT1***	Serine hydroxymethyltransferase 1	11.19	0.0091
**SERPING1***	serpin family G member 1	15.03	0.0126
**APOC3***	apolipoprotein C3	15.72	0.0062
**ALDH3A1***	Aldehyde Dehydrogenase 3 Family Member A1	17.69	0.0055
