# Supplementary material for: Proteomic Profiling of Tears in Blau Syndrome Patients in Identification of Potential Disease Biomarkers
Source: Int J Mol Sci. 2024 Aug 1;25(15):8387. doi: 10.3390/ijms25158387 (PMC11312868; doi:10.3390/ijms25158387)
Supplement: Supplementary file 1 [file ijms-25-08387-s001.zip › Table S6.pdf]

Table S6: Significantly differentially abundant proteins between I1 new sample and I1 old sample. Protein with a Fold Change (FC) > |2| are reported; protein with the FC > |5| (in bold) should be of interest.

Gene name	Protein description	fold change	p-value
**KRT4**	**Keratin 4**	-7.27	0.0252
KRT13	Keratin 13	-4.18	0.0208
SOD2	Superoxide Dismutase 2	-3.82	0.0030
KRT1	Keratin 1	-3.03	0.0018
KRT5	Keratin 5	-2.83	0.0362
KRT16	Keratin 16	-2.73	0.0007
KRT7	Keratin 7	-2.57	0.0073
ATP5F1A	ATP synthase F1 subunit alpha	-2.37	0.0074
KRT6C	Keratin 6C	-2.12	0.0296
H4C1	H4 clustered histone 1	-2.03	0.0013
KRT19	Keratin 19	2.02	0.0487
Krt5	Keratin 5	2.66	0.0075
KRT10	Keratin 10	2.78	0.0089
KRT9	Keratin 9	2.86	0.0114
FGA	Fibrinogen alpha chain	3.06	0.0102
VTN	Vitronectin	3.06	0.0002
KPRP	Keratinovyte proline rich protein	4.51	0.0114
**KRT14**	**Keratin 14**	8.72	0.0060
